# A new species of *Trite* Simon, 1885 (Araneae: Salticidae) from New Zealand, with remarks on *Trite* relationships and radiation

**DOI:** 10.7717/peerj.3463

**Published:** 2017-06-20

**Authors:** Barbara Patoleta, Marek Żabka

**Affiliations:** Department of Zoology, Faculty of Natural Sciences, Siedlce University of Natural Sciences and Humanities, Siedlce, Poland

**Keywords:** Jumping spiders, Taxonomy, Biogeography, Island radiation

## Abstract

A species known from earlier behavioural studies as “*Holoplatys sp*.”, is described as *Trite pollardi* sp. nov. Within the genus *Trite*, two species groups are distinguished: the *planiceps*-group (found in New Caledonia, New Zealand, Lord Howe Island and Norfolk Island) and the *incognita*-group (limited to New Zealand). The three alternative scenarios of the *Trite* origin, relationships and radiation in New Zealand, New Caledonia and Lord Howe Island are discussed. Three species are considered to be excluded from *Trite*.

## Introduction

There are some 200 salticid species predicted to occur in New Zealand (Żabka, Pollard & Anstey, 2002), but only 50 of them are formally described (Żabka & Pollard, 2002b; Sirvid et al., 2011) and less than 10 species are recognisable (Bryant, 1935; Żabka, 1988; Żabka, 2004; Żabka & Pollard, 2002a; Żabka & Pollard, 2002c; Vink, Duperre & McQuillan, 2011). Other nominal species are poorly documented, misplaced in wrong genera or published as *nomina dubia* (see review in: Paquin, Vink & Dupérré, 2010; Sirvid et al., 2011).

A spider known as “*Holoplatys* sp.” was subjected to behavioural studies (Jackson & Harding, 1982), but its biology raised some doubts about its generic placement. Instead of being a typical tree trunk and bark dweller (see Żabka, 1991b), “*Holoplatys* sp.” was found in a variety of microhabitats, including vegetation, mainly New Zealand flax (*Phorbium tenax*: family Asphodelaceae). During MŻ’s research in New Zealand in 2000 the opportunity was taken to clarify the case. As suspected, the species was not *Holoplatys*, but belonged in the genus *Trite*—well known from New Zealand and other island groups in South West Pacific (Table 1). Further studies revealed many other undescribed species of *Trite* and inspired us to investigate the genus’ origin and relationships.

**Table 1:** The verified list of *Trite* species and their distribution.

Valid species	NZ	NC	LH, NI	O
1. *Trite caledoniensis*Patoleta, 2014		+		
2. *Trite concinna* Rainbow, 1920			+	
3. *Trite gracilipalpis* Berland, 1929				+
4. *Trite grayi*Richardson, 2016			+	
5. *Trite guilberti*Patoleta, 2014		+		
6. *Trite herbigrada* (Urquhart, 1889)	+			
7. *Trite lineata*Simon, 1885		+		
8. *Trite longipalpis* Marples, 1955				+
9. *Trite mustillina* (Powell, 1873)	+			
10. *Trite parvula* (Bryant, 1935)	+			
11. *Trite pennata*Simon, 1885		+		
12. *Trite planiceps* Simon, 1899	+			
13. *Trite ponapensis*Berry, Beatty & Prószyński, 1997				+
14. *Trite simoni*Patoleta, 2014		+		
15. *Trite urvillei* (Dalmas, 1917)	+			
**Species to be excluded from*Trite***				
1. *Trite auricoma* (Urquhart, 1886)	+			
2. *Trite ignipilosa* Berland, 1924		+		
3. *Trite rapaensis* Berland, 1942				+

**Notes.**

NZ New Zealand NC New Caledonia LHI Lord Howe Island NI Norfolk Island O Other areas: Tonga, Samoa, Caroline and Loyalty Islands

## Material & Methods

The specimens for study came from the following collections: Auckland Museum Entomology Collection—AMNZ; Canterbury Museum, Christchurch—CMNZ; Entomology Research Museum, Lincoln—LUNZ; Museum of New Zealand, Wellington—MONZ; New Zealand Arthropod Collection, Auckland—NZAC; Otago Museum, Dunedin—OMNZ.

The examination methods were as described by Żabka (1991a). The drawings were made using a grid system. The photographs were taken with Nikon D5200 camera and Nikon SMZ1000 stereomicroscope, and were digitally processed with ZoomBrowser and HeliconFocus software. The actual and predicted distributional maps were generated with DIVA-GIS bio-climatic software using BIOCLIM application (Nix, 1986; Busby, 1991). Our model has been produced with 12 field records and met the requirements for the software (at least 5–10 records; Hernandez et al., 2006). The following environmental variables were used in the analysis: annual mean temperature, mean monthly temperature range, isothermality, temperature seasonality, max temperature of warmest month, min temperature of coldest month, temperature annual range, mean temperature of wettest quarter, mean temperature of driest quarter, mean temperature of warmest quarter, mean temperature of coldest quarter, annual precipitation, precipitation of wettest month, precipitation of driest month, precipitation seasonality, precipitation of wettest quarter, precipitation of driest quarter, precipitation of warmest quarter, precipitation of coldest quarter.

The origin and relationships of *Trite*, were considered in the light of the available morphological and molecular data (Maddison, Bodner & Needham, 2008; Bodner & Maddison, 2012; Maddison, 2015).

The electronic version of this article in Portable Document Format (PDF) will represent a published work according to the International Commission on Zoological Nomenclature (ICZN), and hence the new names contained in the electronic version are effectively published under that Code from the electronic edition alone. This published work and the nomenclatural acts it contains have been registered in ZooBank, the online registration system for the ICZN. The ZooBank LSIDs (Life Science Identifiers) can be resolved and the associated information viewed through any standard web browser by appending the LSID to the prefix http://zoobank.org/. The LSID for this publication is: urn:lsid:zoobank.org:pub:EC2FDD31-400F-4221-A96C-D48E1BDB684A. The online version of this work is archived and available from the following digital repositories: PeerJ, PubMed Central and CLOCKSS.

## Results and Discussion

### Genus *Trite* Simon, 1885

*Trite* Simon, 1885: 91, 1903: 829; Dalmas, 1917: 420; Roewer, 1954: 1031; Bonnet, 1959: 4694; Berry, Beatty & Prószyński, 1997: 132; Żabka & Pollard, 2002b: 77; Vink, Duperre & McQuillan, 2011: 317; Patoleta, 2014: 359; Richardson, 2016: 552; Prószyński, 2016.

**Type species**: *Trite pennata* Simon, 1885 (New Caledonia), subsequently designated by Simon (1903).

### Remarks on relationships and radiation

**Figure 1:**
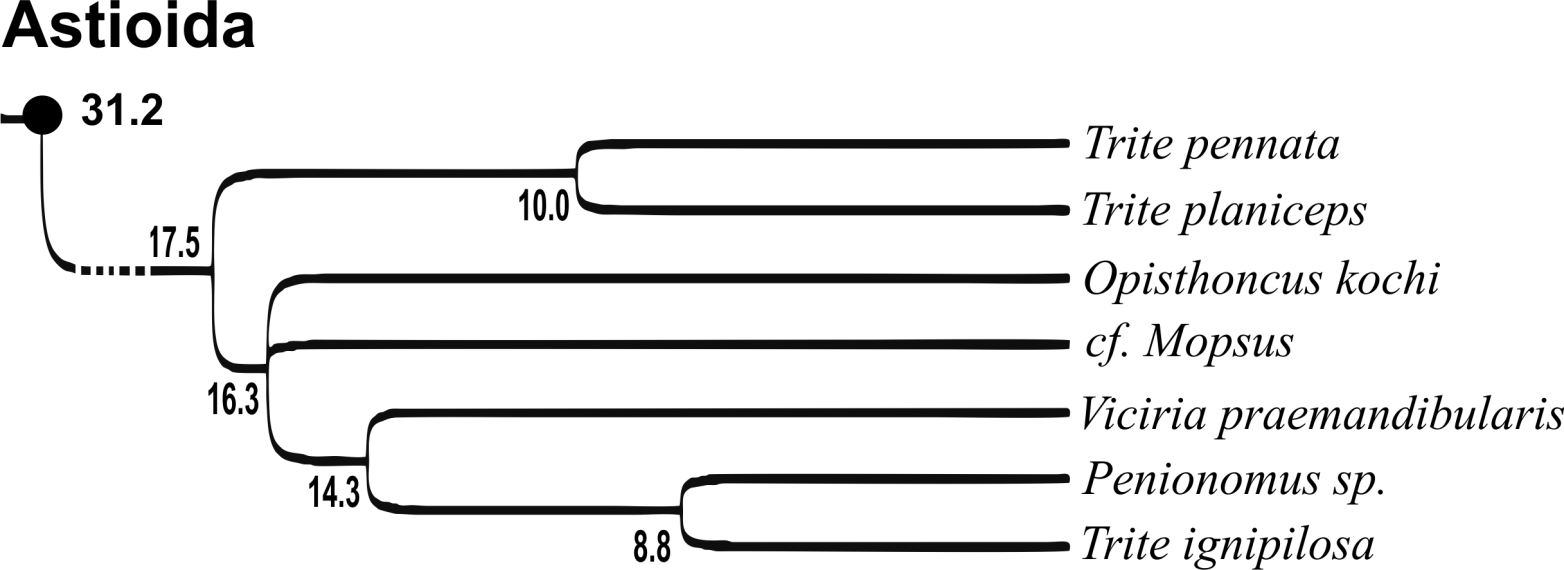
Time-calibrated phylogeny (after Bodner & Maddison, 2012, modified), numbers beside nodes are estimated ages in millions of years.

**Figure 2:**
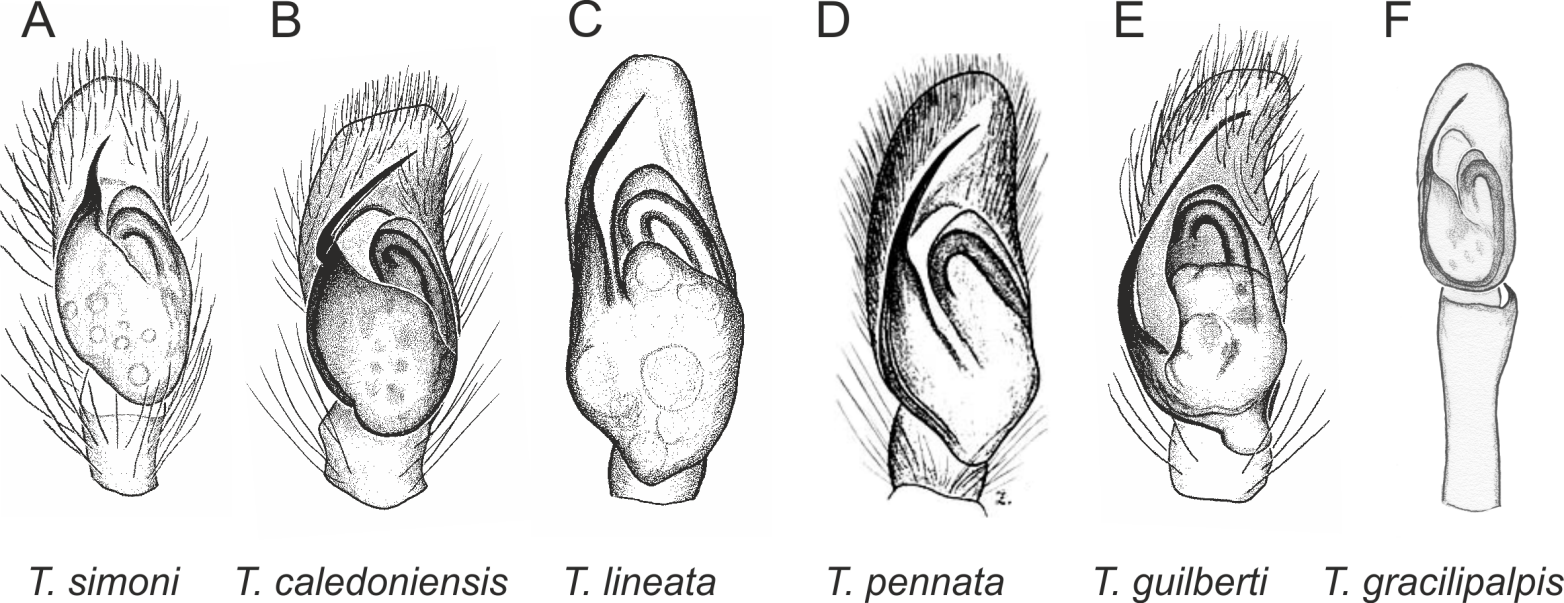
Palpal organ variety in *Trite* species (*planiceps*-group) from New Caledonia and Loyalty Islands.

**Figure 3:**
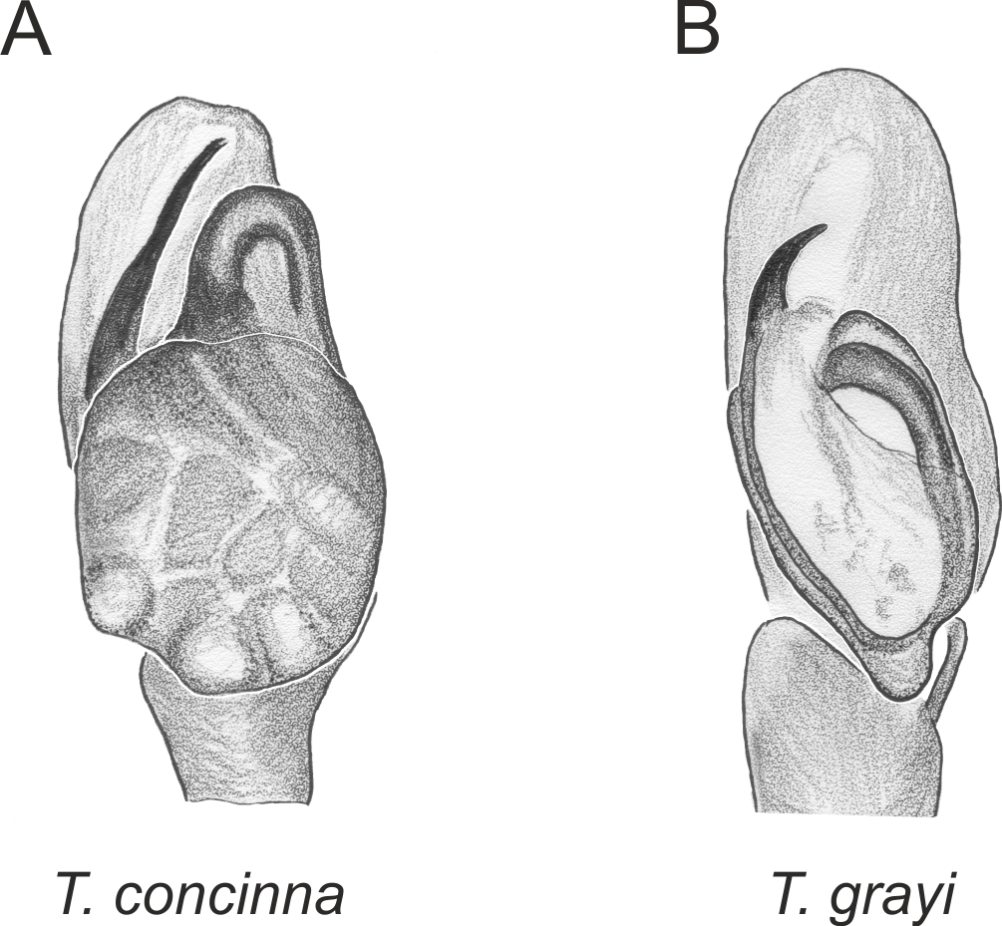
Palpal organ variety in *Trite* species (*planiceps*-group) from Lord Howe and Norfolk Islands.

**Figure 4:**
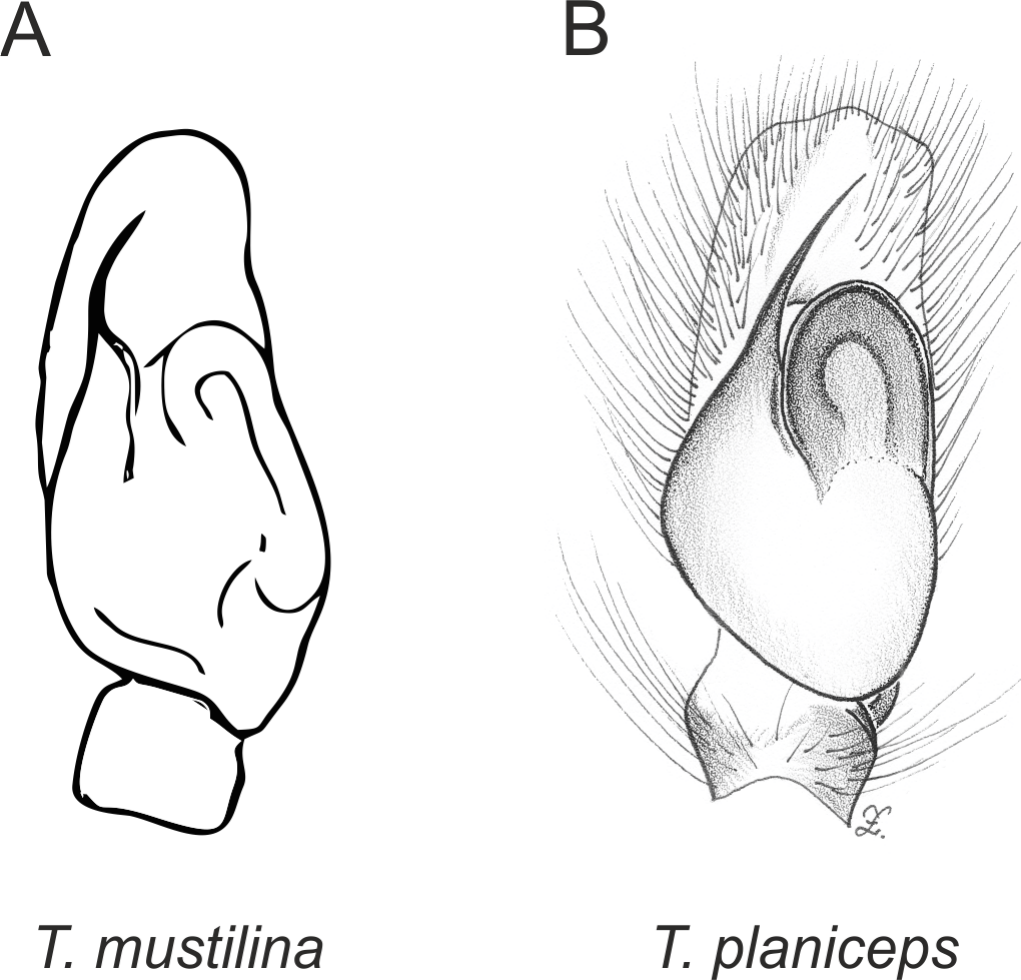
Palpal organ variety in *Trite* species (*planiceps*-group) from New Zealand ((A) after Bryant, 1935, modified).

**Figure 5:**
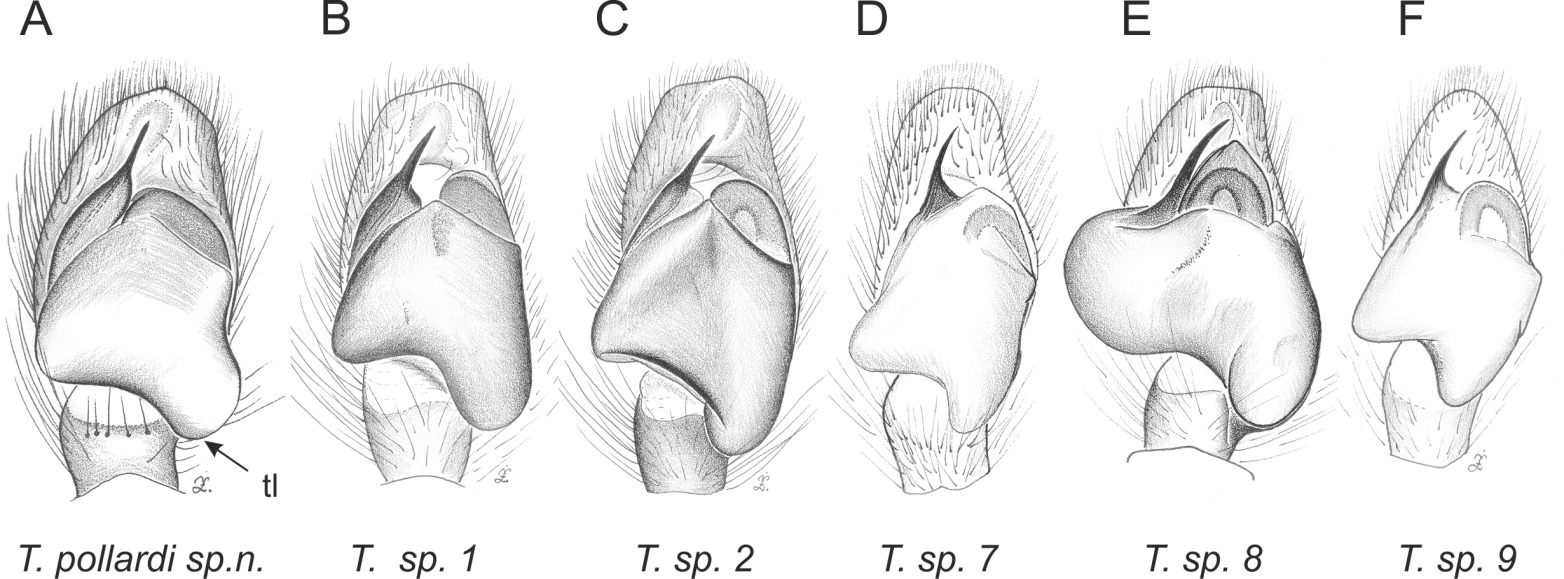
Palpal organ variety in *Trite* species (*incognita*-group) from New Zealand; tl, tegular lobe is more distinctive and tegulum is wider than in the *planiceps*-group.

**Figure 6:**
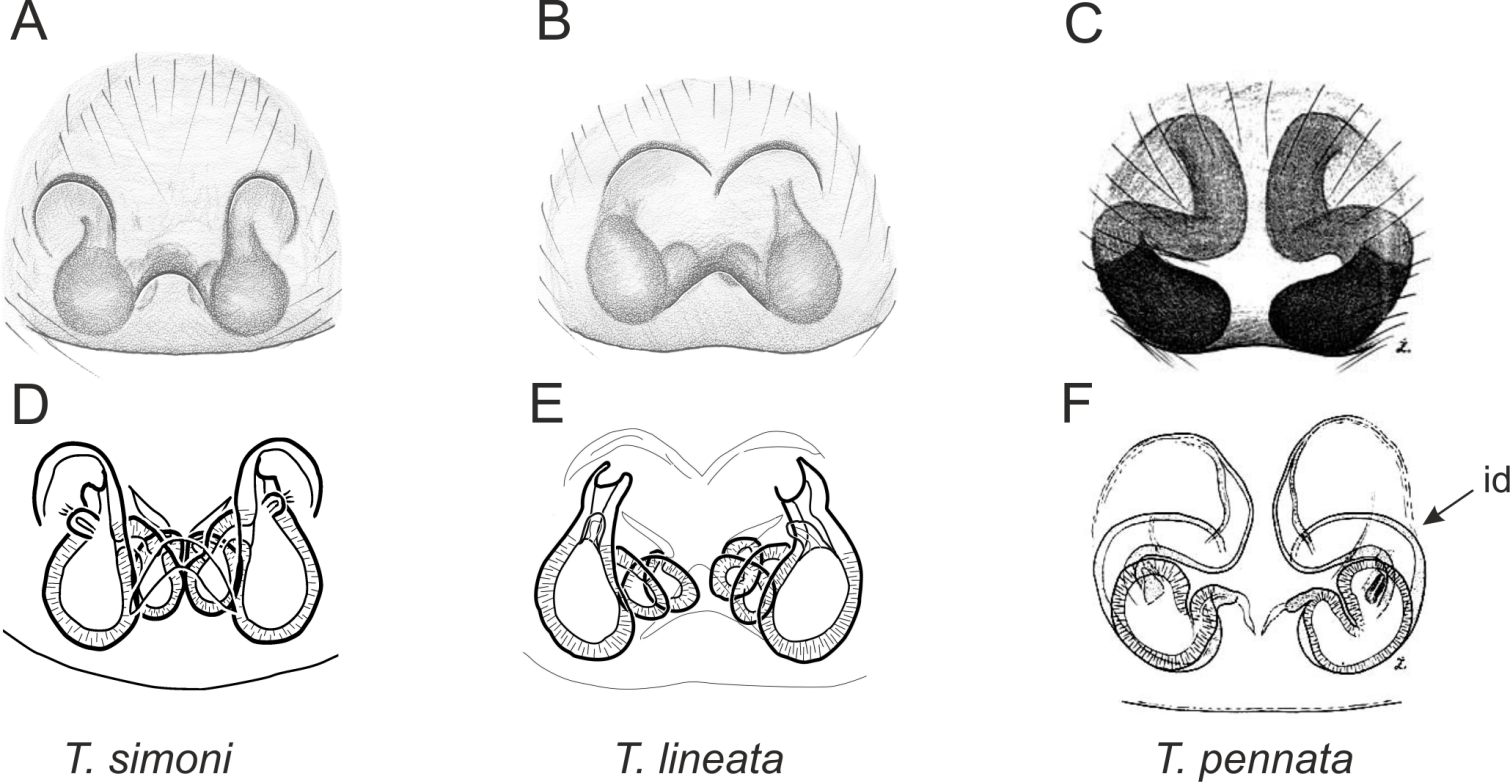
Epigyne and internal genitalia in *Trite* species (*planiceps*-group) from New Caledonia; id, insemination duct; distinctive for the group.

**Figure 7:**
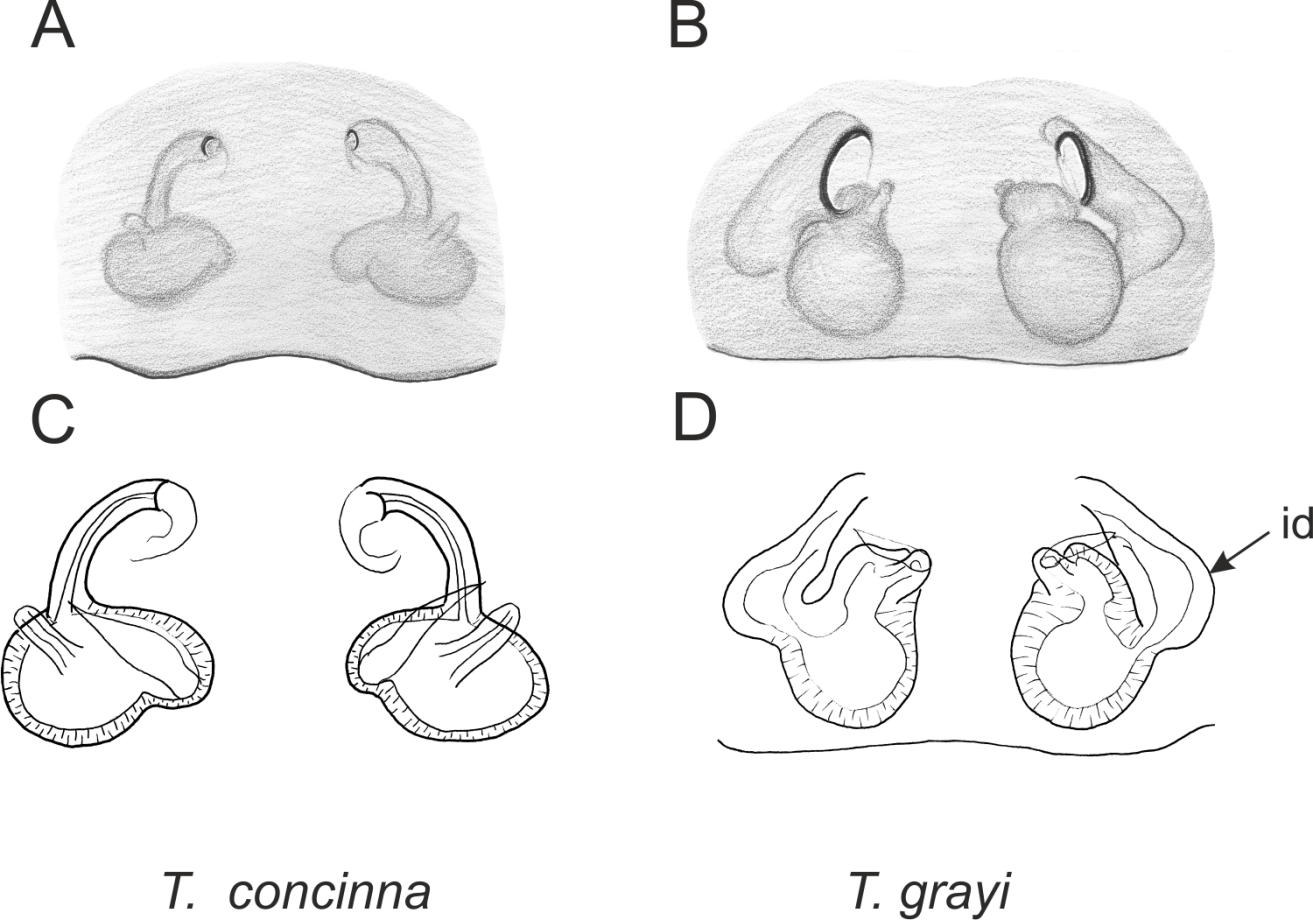
Epigyne and internal genitalia in *Trite* species (*planiceps*-group) from Lord Howe and Norfolk Islands; id, insemination duct; distinctive for the group.

**Figure 8:**
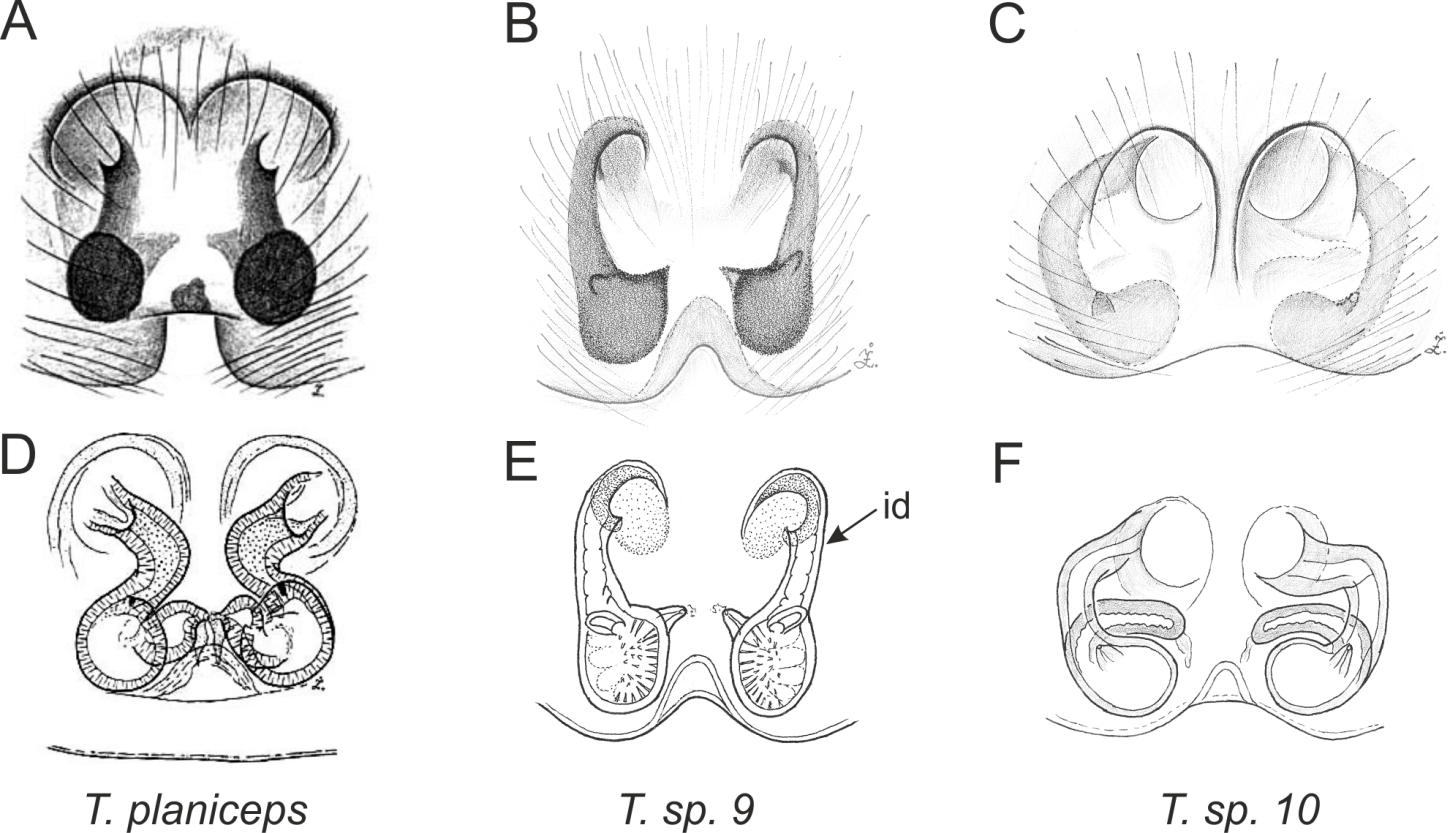
Epigyne and internal genitalia in *Trite* species (*planiceps*-group) from New Zealand; id, insemination duct; distinctive for the group.

**Figure 9:**
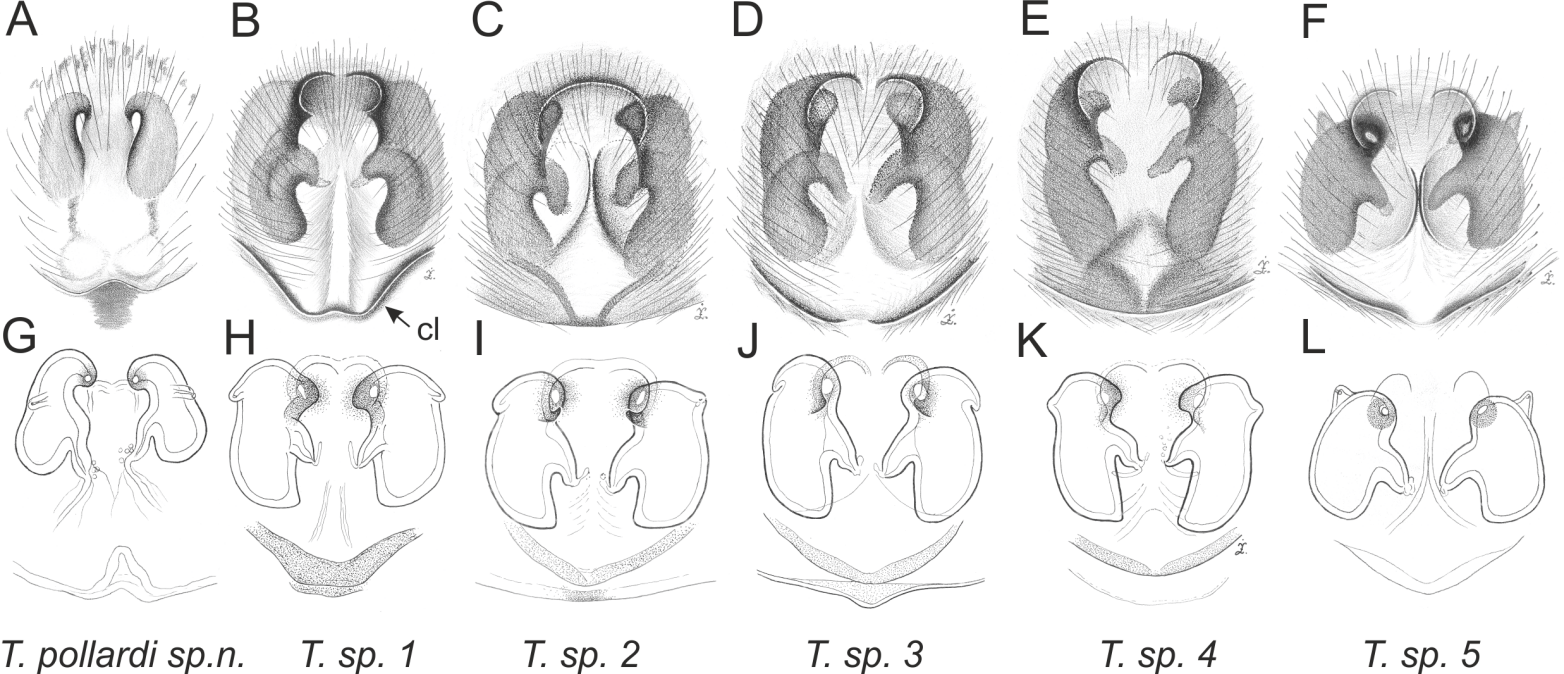
Epigyne and internal genitalia in *Trite* species (*incognita*-group) from New Zealand; insemination ducts are virtually missing, and caudal lobe (cl) is distinctive for the group.

Of the 18 nominal species of *Trite* (World Spider Catalog, 2017) (Table 1), three are non congeneric, two of them *T*. *ignipilosa* and *T*. *rapaensis* requires type material and their generic placement will be decided in separate paper, while “*T*. *auricoma*” and related (undescribed) species are another example of intense radiation in New Zealand; for all of them a new genus should be established. Others occur in New Zealand (5 spp.), New Caledonia (5 spp.), Lord Howe Island (2 spp.) and on smaller island groups (Żabka, 1988; Berry, Beatty & Prószyński, 1997; Patoleta, 2002; Patoleta, 2014; Richardson, 2016). According to molecular data, the genus *Trite* is part of the Australasian Astioida clade and Vicirini tribe, the latter being dominated by Australian and New Caledonian genera (Maddison, Bodner & Needham, 2008; Maddison, 2015). The whole Astioida clade arose ∼30 MYA, while the genus *Trite* is ∼10 MYA (*T. pennata* from New Caledonia and *T. planiceps* Simon, 1899 from New Zealand) (Fig. 1) (Bodner & Maddison, 2012; Zhang & Maddison, 2013). The analysis of genitalic structures in *Trite* allowed us to distinguish two species groups (Figs. 2–9).

In the *planiceps*-group (Figs. 2–4, Figs. 6–8) the male palpal tegulum is narrow, about the width of the cymbium, without or with a small tegular lobe. The female epigyne is not especially elongate, and without a caudal lobe. The internal genitalia are close to the epigastric furrow, the insemination ducts distinctive and spermathecae ovoid or pear-shaped. The group is found in New Caledonia, New Zealand, Lord Howe Island and Norfolk Island.

**Figure 10:**
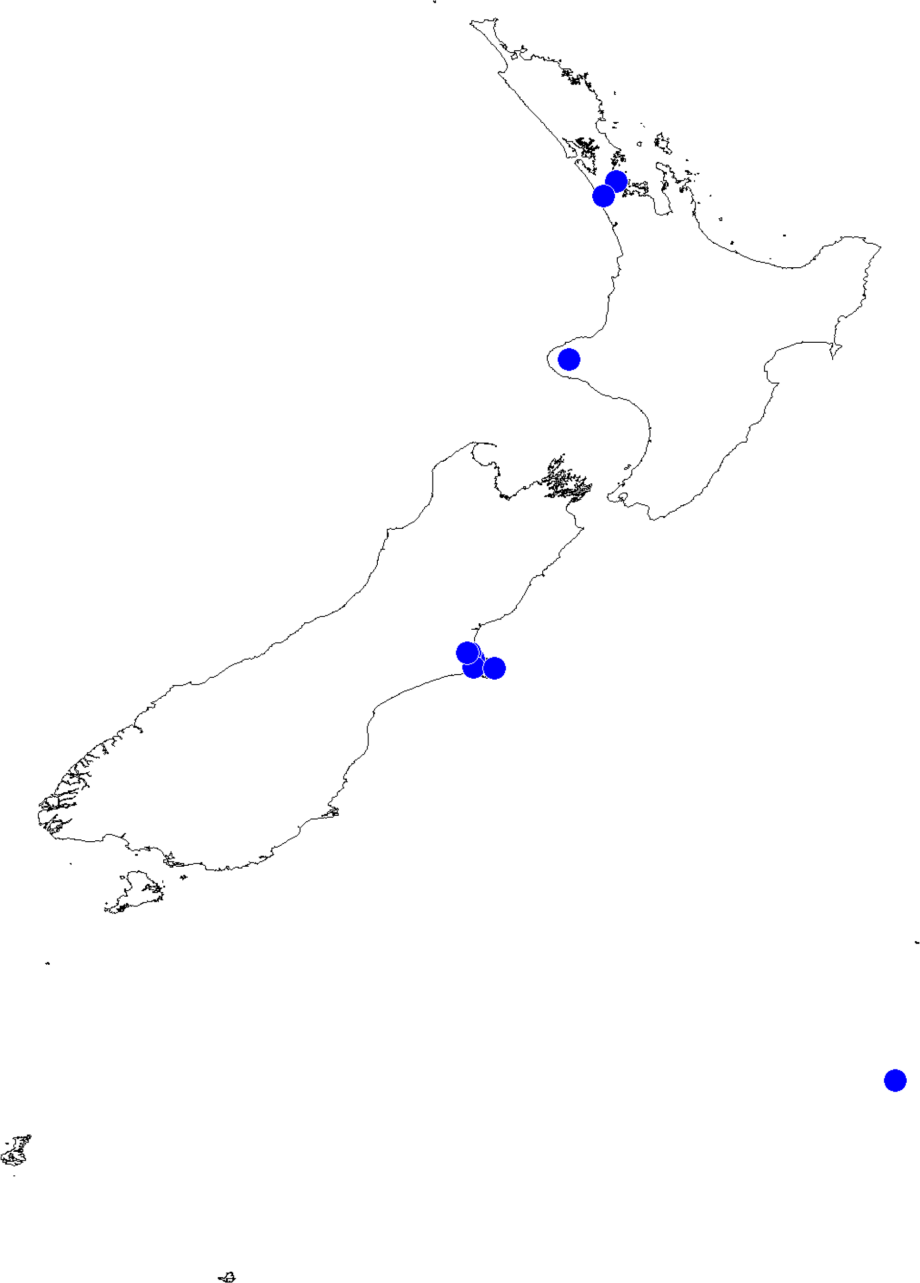
*Trite incognita*-group distribution records in New Zealand.

In the *incognita*-group (Figs. 5 and 9) the tegulum is robust (trapezoid), wider than the cymbium and with a distinctive lobe (tl). The epigyne is elongate, with a caudal lobe (cl). The internal genitalia are distant from the epigastric furrow, spermathecae are large and pear-shaped and insemination ducts are almost missing. Particular species within the group differ in tiny details proving they are the result of a very recent radiation. We consider this pattern more derived. The group is limited to New Zealand (Fig. 10).

To discuss the history of *Trite*, it is necessary to consider geological and climatic circumstances for the area of *Trite* occurrence.

Until quite recently, the nature of New Zealand and New Caledonia was often discussed in terms of Gondwanan heritage, but geological data show much greater complexity and are only partly supportive of this view. After separation from Gondwana (∼85 MYA), both island groups (New Zealand and New Caledonia) were part of the Zealandia land mass (e.g., Cluzel et al., 2012; Campbell et al., 2012). Since the late Cretaceous, New Caledonia has experienced many episodes of subduction and submergence and emerged only in post-Eocene, 37 MYA (Cluzel et al., 2012). The fate of New Zealand was even more dramatic (Campbell et al., 2012; Sharma & Wheeler, 2013). The interactions between the Pacific and Australian plates (30 MYA) initiated multiple submergences of large parts (but possibly not all) of New Zealand (Mildenhall et al., 2014). Over the last 5 MY, the events intensified (Cox & Findlay, 1995; Batt et al., 2000), resulting in the uplift of the Southern Alps and severe Pleistocene climate changes. All these circumstances might have caused multiple extinctions and stimulated speciation and local radiations. Given the timing proposed by Bodner & Maddison (2012), the salticid fauna of both island groups should be considered as the result of (1) colonisations from other sources, mainly from Australia over the last 30 MY and (2) local radiation.

Lord Howe Island is located on the submerged Lord Howe Rise (Mortimer et al., 2017), which links the island to New Caledonia. The present Lord Howe Island is dated as 7 MYA—old enough to develop some endemic species, but too young and small (18.576 km^2^) to expect a high diversity of biota and higher-level endemic taxa (genera). The Lord Howe Island salticids probably derived from Australian and New Caledonia ancestors, which reached the islands by dispersal.

The above circumstances allow us to propose three alternative scenarios for *Trite* radiation.

**1. New Caledonia as the origin for** ***Trite***: strongly supported by the presence of *planiceps*-group of species, but, first of all, by several related astioid genera (*Corambis* Simon, 1901, *Penionomus* Simon, 1903, *Rhondes* Simon, 1901*, Lystrocteisa* Simon, 1884, *Rogmocrypta* Simon, 1900), all endemic for New Caledonia with relatives (sister genera?) in Australia (Patoleta, 2002; Patoleta, 2017; Bodner & Maddison, 2012). Under this scenario the first New Caledonia Astoida arrived from Australia some 30 MYA. The genus *Trite* appeared ∼10 MYA, radiated within the *planiceps*-group and spread to New Zealand (*T*. *planiceps*, *T. pennata*). The species in Lord Howe Island, Norfolk or other islands would be the result of dispersal and radiation *in situ*.

**2**. **New Zealand as the origin**: supported by far the largest number of species in both groups and the *incognita*-group being limited to New Zealand. However, the lack of ancestral (related) genera makes this scenario less likely. The diversity within the *incognita-* group suggests recent local radiation within the last 5 MY. Such a possibility is also supported by New Zealand Lycosidae (Vink & Paterson, 2003) and Thomisidae (Sirvid et al., 2013).

**3. Australia as the source of** ***Trite*** **ancestors**: supported by the similarities in genitalic structures between the *planiceps*-group and some Australian genera (*Pungalina* Richardson, 2013) (Richardson, 2013; Richardson, 2016).

Because the molecular data are only available for *T*. *ignipilosa*, *T*. *pennata* and *T*. *planiceps*, at this stage we can only rely on genital morphology, geological factors and generic-level classification proposed by Maddison (2015). The above scenarios can only be verified by complete molecular data for all species groups.

**Figure 11:**
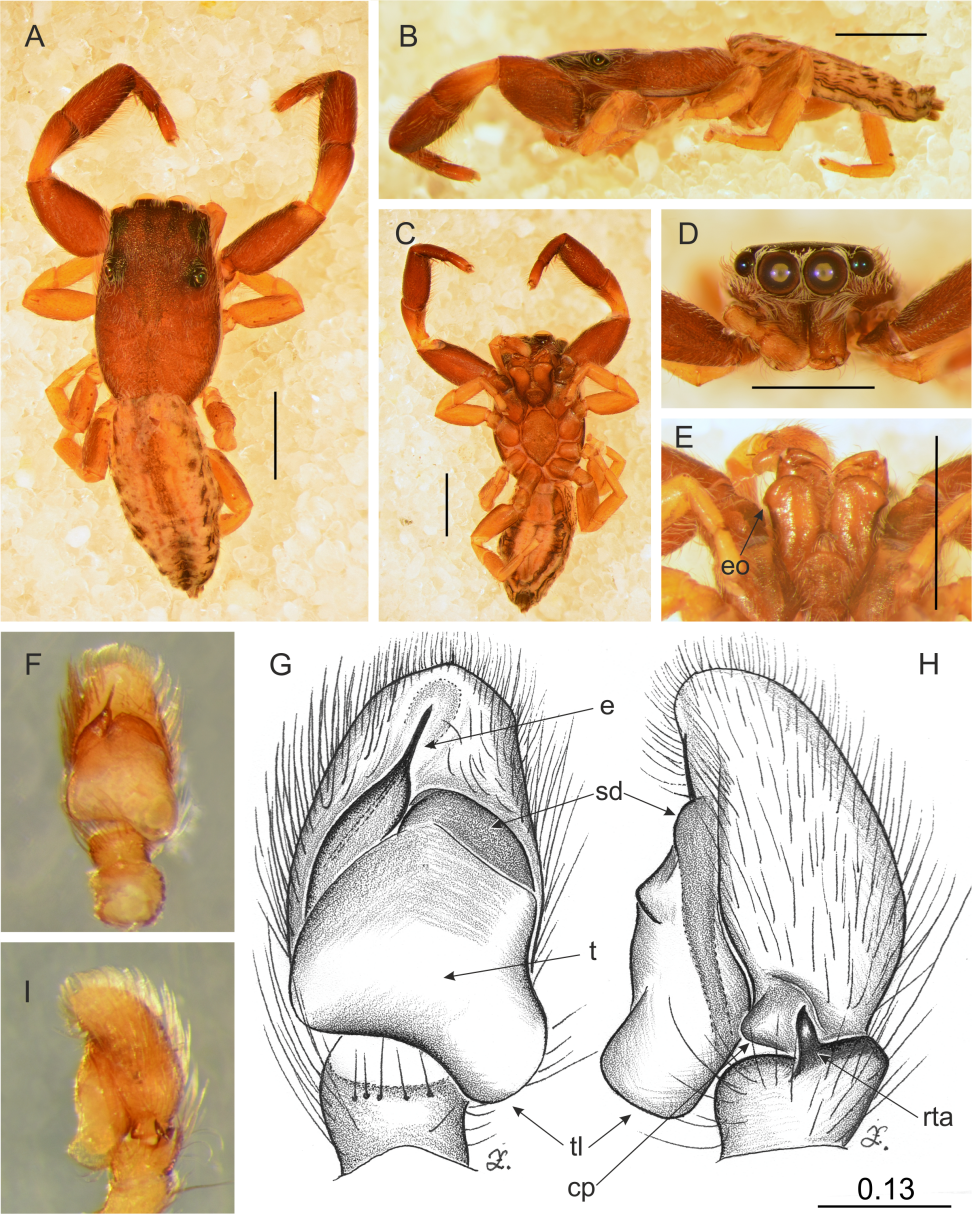
*Trite pollardi* sp. n. (holotype). (A) Dorsal view (B) lateral view (C) ventral view (D) frontal view (E) Endites and labium (F–G) Left palp ventrally (H–I) same, retrolaterally. Scale bar: Figs (A–E) = 1 mm, (G–H) as in fig.

**Figure 12:**
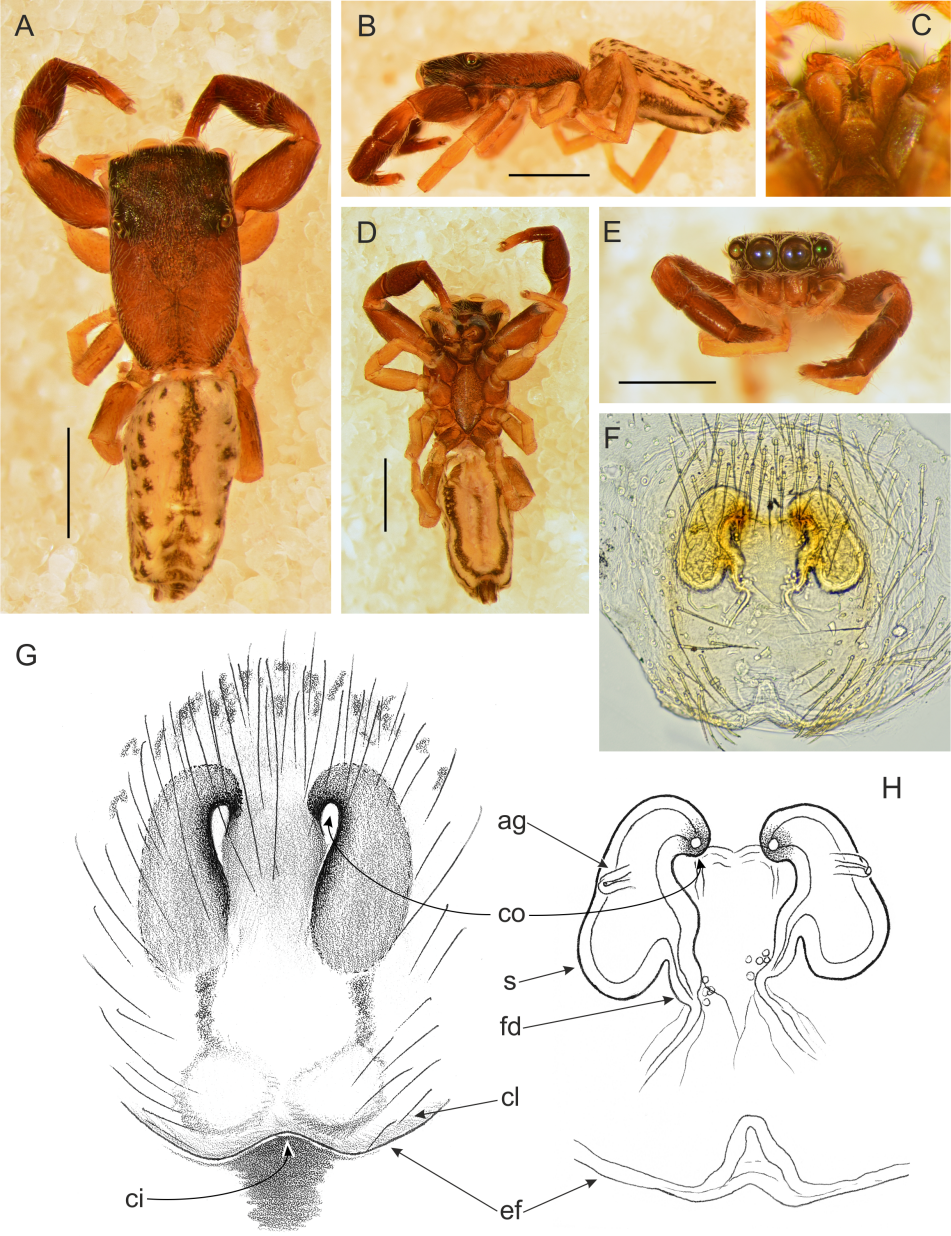
*Trite pollardi* sp. n. (paratype). (A) Dorsal view (B) lateral view (C) Endites and labium (D) ventral view (E) frontal view (F, H) vulva (G) epigyne. Scale bar: Figs (A–E) = 1 mm.

***Trite pollardi*** sp. nov.

Figs. 11 and 12

**Holotype**

1♂, Lake Rotoiti, 42.4°S 173.6°E, under manuka bark, 25.01.1949, Cawthron, NZAC.

**Figure 13:**
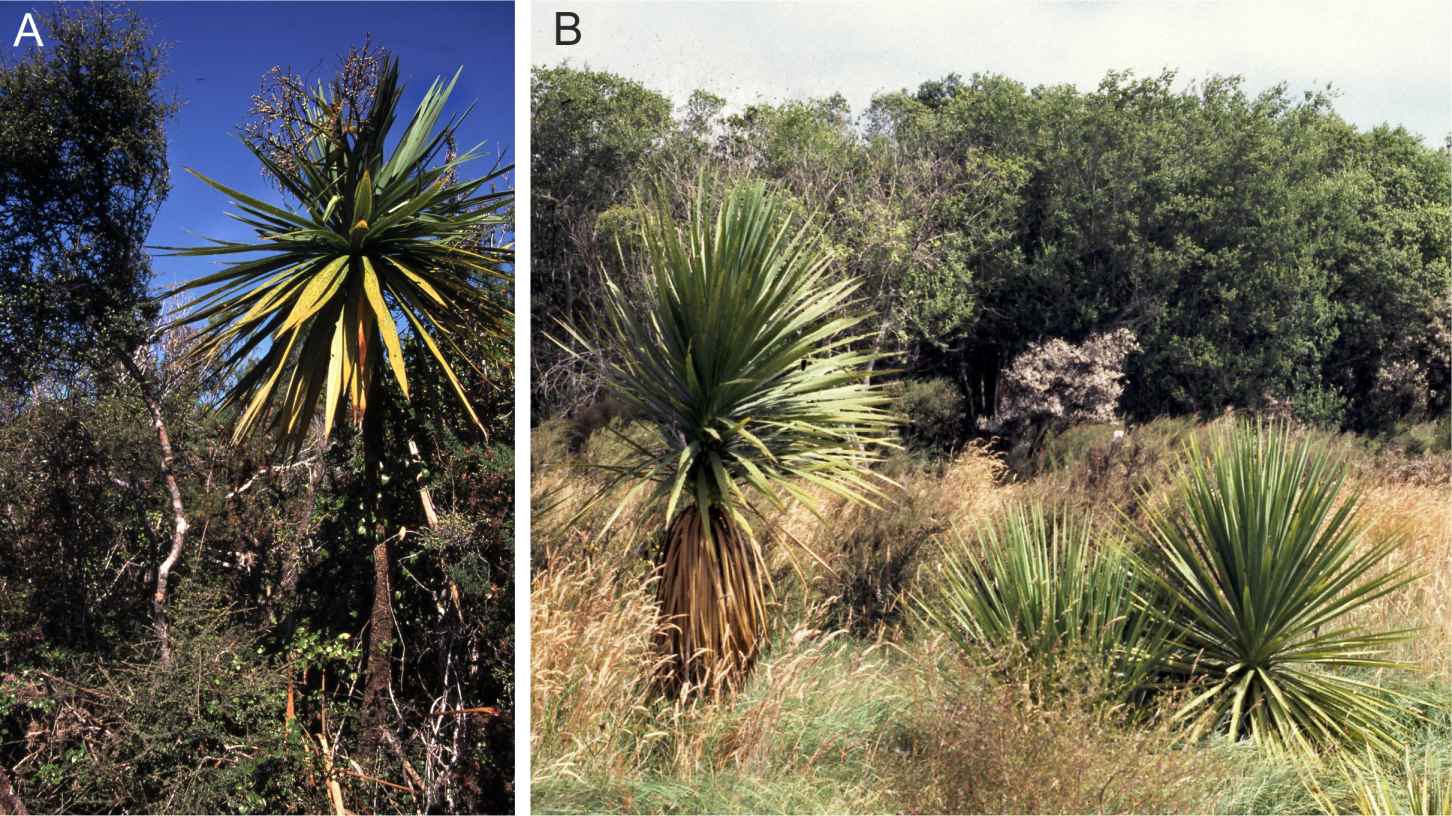
*Trite pollardi* sp. n. habitats (Photograph by MŻ).

**Figure 14:**
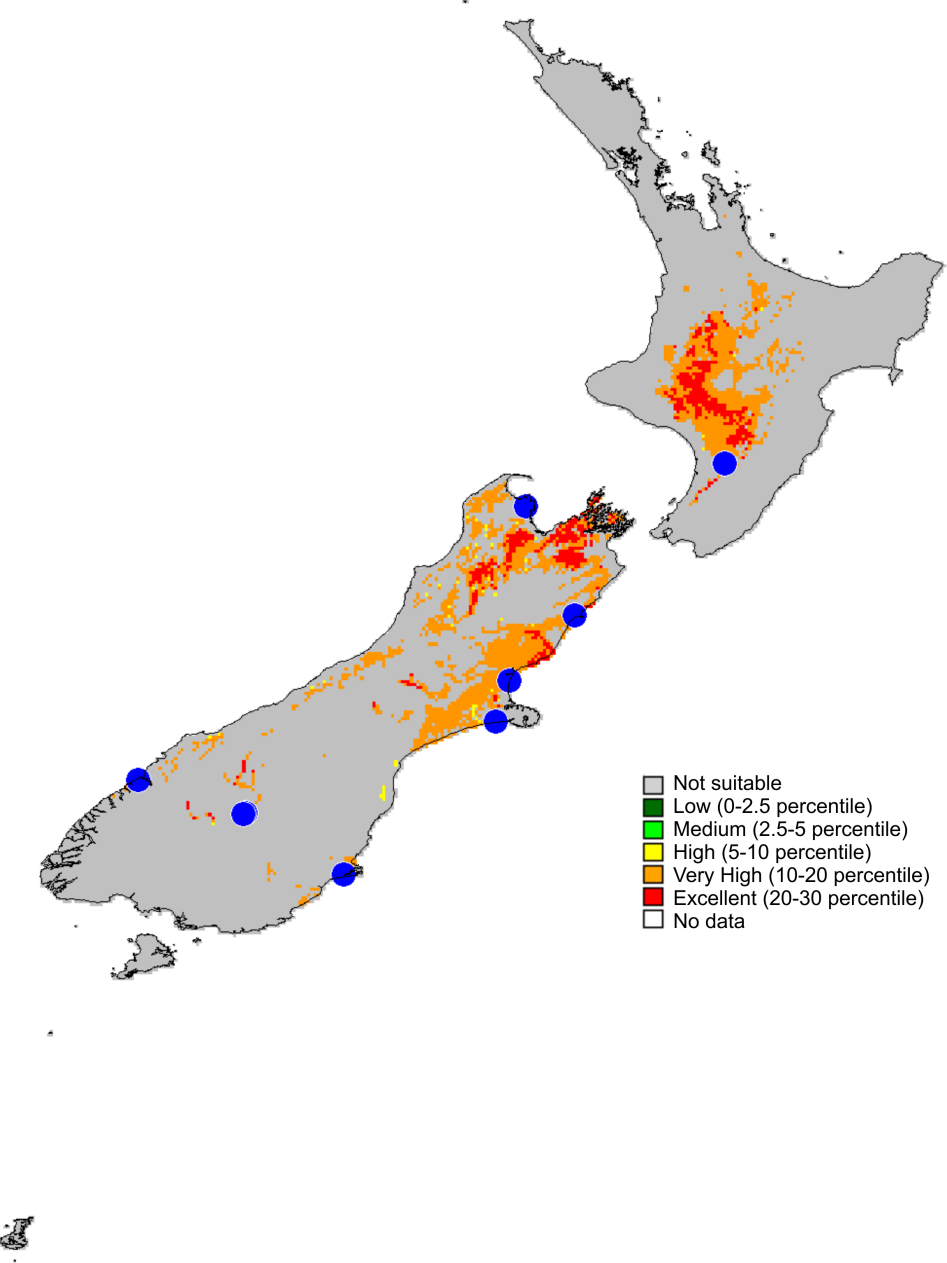
Recorded localities and predicted distribution of *Trite pollardi* sp. n.

**Paratypes**

2♀, same data as holotype; 1♂, 1♀, Otago, Westwoos, 45.8833°S, 170.5°E, sand dunes, December 1990, Murphy, nr 20125, OMNZ; 1♂, Porters Pass, 40.9333°S, 172.95°E, under rocks near river, 19.01.2000, MP Anstey, CMNZ; 1 ♀, Waikuku Beach 43.2833°S, 172.7167°E, ex Marram grass [*Ammophyla* sp.], 14.09.1971, KM Mason, LUNZ; 1♂, 2 juv., Cromwell, Cromwell Chafer Beetle Nature Reserve, Cemetery Rd end, Tussock litter, 17.11.1977, JC Watt, NZAC; 1♀, Kaitorete Spit, MC, Banks Peninsula, 43.8283°S 172.5401°E, in cracks in driftwood, with egg sac, 01.11.1991, CJ Vink, LUNZ; 1♀, Flax Swamp, Spencer Park, 44.6167°S 167.7333°E, in rolled flax, 03.02.2000, S Pollard, CMNZ; 1♀, Kawarau Gorge, 45.0667°S 169.15°E, 17.11.1977, JC Watt, NZAC; 1♀, Kaitorete Spit, Birdlings Flat, 43.8283°S 172.5401°E, under stones near beach, 06.03.1999, H Ranson, LUNZ; 3♂, 2♀, 3 juv., Cromwell Beatle Reserve, Cemetery Rd end, 45.054914°S, 169.169152°E, Tussock & litter, 17.11.1977, JC Watt, NZAC; 2♂, Old Man Range, Symes Rd, 45.3667°S 169.2167°E, litter, 1372 m, 06.02.1983, JC Watt, NZAC; 2♂, 2 ♀, Bobby’s Head Rd near Palmerston, 40.3549°S 175.6095°E, tussock, 20.10.1966, CL Wilton, OMNZ.

**Etymology**. For Dr. Simon Pollard, New Zealand arachnologist and author of many papers on spider behaviour.

**Diagnosis**. Differs from other species of *Trite* by the following combination of characters: body flat (*Holoplatys*-like), lighter abdominal colour pattern, palpal organ with a distinctive tegular lobe, cymbium with protuberance, epigyne caudal lobe with median incision, copulatory openings accompanied by sclerotized crevice, fertilization ducts narrow, close to the epigastric fold.

**Description**. Male holotype (Fig. 11). Body flat and narrow. Cephalothorax much longer than wide, with gentle posterior slope, brown, covered with sparse white scales. Eye field wider than long, its length 35% of CL, PME closer to ALE than PLE. Fovea located well behind the eye field. Abdomen pale with darker central stripe and lateral spots, covered with sparse brown hairs. Anterior margin with protruding white hairs. Clypeus brown, very narrow, 9% of AME diameter, covered with long, white hairs. Chelicerae brown, slender, with white scales more numerous at the base. Promargin with two teeth, retromargin with one tooth. Endites slender, with lateral outgrowth, brown, anteriorly lighter. Labium brown. Sternum longer than wide, light brown. Venter whitish with grey-brown lateral band. Spinnerets grey. Palps light brown, cymbium with lighter tip. Tibia short, RTA short with slightly hooked tip. Tegulum longer than wide, with large tegular lobe. Embolus thin, arising from a distinct mound on the anterior distal edge of tegulum. Legs I the strongest, with swollen femora and tibiae, brown. Metatarsi with proventral (1–1) and retroventral (1–1) spines. Tibiae with one prolateral spine. Other legs light brown. Leg formula: 1-4-2-3. Dimensions. CL 2.34, CW 1.41, CH 0.49, AL 2.30, AW 1.17, EFL 0.82, AEW 1.10, PEW 1.14, L I: 5.08, L II: 3.15, L III: 2.77, L IV: 3.74.

Female paratype (Fig. 12). Similar to the male, but clypeal hairs less numerous and shorter, chelicerae much shorter and protruding hairs on anterior margin of abdomen absent. Epigyne with caudal lobe. Copulatory openings oriented backwards and situated anteriorly, far from the epigastric furrow. Separate insemination ducts missing. Spermathecae pear-shaped, with accessory glands on lateral walls. Fertilisation ducts well marked. Dimensions. CL 1.96, CW 1.18, CH 0.41, AL 2.07, AW 1.07, EFL 0.67, AEW 1.08, PEW 1.06, L I: 3.63, L II: 2.64, L III: 2.31, L IV: 3.35.

**Biology**. The species is usually found on New Zealand flax (Fig. 13) and tussock grass, but also occasionally under rocks, in crevices of driftwood and under manuka bark (*Leptospermum scoparium*: family Myrtaceae). *Trite pollardi* has large repertoire mating behaviours (Jackson & Harding, 1982).

**Distribution** (Fig. 14). The species has been recorded all over the North and South Islands of New Zealand.

## Abbreviations

AEW: Anterior eye width
ag: Accessory gland
AME: Anterior medial eyes
AL: Abdomen length
AW: Abdomen width
CH: Cephalothorax height
ci: Caudal icision
CL: Cephalothorax length
cl: Caudal lobe
co: Copulatory opening
cp: Cymbial protuberance
CW: Cephalothorax width
EFL: Eye field length
e: Embolus
ef: Epigastric furrow
eo: Endite outgrowth
fd: Fertilization duct
id: Insemination duct
L: Leg
PEW: Posterior eye width
PLE: Posterior lateral eyes
PME: Posterior medial eyes
rta: Retrolateral tibial apophysis
sd: Sperm duct
t: Tegulum
tl: Tegular lobe

## Additional Information and Declarations

### Data Availability

The following information was supplied regarding data availability: Locations used to draw maps are included as Holotype and Paratype material in the manuscript. The specimens for study came from the following collections: Auckland Museum Entomology Collection; Canterbury Museum, Christchurch; Entomology Research Museum, Lincoln; Museum of New Zealand, Wellington; New Zealand Arthropod Collection, Auckland; Otago Museum, Dunedin.

### New Species Registration

The following information was supplied regarding the registration of a newly described species: Publication LSID: urn:lsid:zoobank.org:pub:EC2FDD31-400F-4221-A96C- D48E1BDB684A Trite pollardi Genus name: urn:lsid:zoobank.org:act:4C93C283-B525-4CB7-B039-7ADDEA74DE62 Species name: urn:lsid:zoobank.org:act:FCD1DF01-DE05-4471-A7ED-C5831405979F.
